# Multidimensional Models of Perfectionism and Procrastination: Seeking Determinants of Both

**DOI:** 10.3390/ijerph17145099

**Published:** 2020-07-15

**Authors:** Allison P. Sederlund, Lawrence R. Burns, William Rogers

**Affiliations:** Psychology Department, Grand Valley State University, Allendale, MI 49417, USA; sederlua@mail.gvsu.edu (A.P.S.); wmrphd@gmail.com (W.R.)

**Keywords:** perfectionism, procrastination, conscientiousness, emotion, fear of failure, temporal orientation, boredom proneness, need for affect, euthymia, time management

## Abstract

Background: Perfectionism is currently conceptualized using a multidimensional model, with extensive research establishing the presence of both maladaptive and adaptive forms. However, the potential adaptability of procrastination, largely considered as a maladaptive construct, and its possible developmental connection to perfectionism remains unclear. The purpose of this study was to examine the individual differences of the multidimensional models of both perfectionism and procrastination, as well as investigating potential links between the two constructs. Methods: A convenience sample of 206 undergraduate students participated in this study. Participants completed a questionnaire consisting of 236 questions regarding the variables under investigation. Results: The adaptive model of procrastination yielded largely insignificant results and demonstrated limited links with adaptive perfectionism, while maladaptive procrastination was consistently associated with maladaptive perfectionism, lending further evidence of a unidimensional model of procrastination. Conclusions: Many previous findings regarding the multidimensional model of perfectionism were replicated, along with new contributions focusing on the dual-process model and temporal orientation.

## 1. Introduction

Perfectionism is characterized by setting exceptionally high standards of performance and unrealistic goals, accompanied by overly critical self-evaluations and concerns over making mistakes [1]. This behavior may not always adversely affect an individual, but in many cases, it leads to the accumulation of transdiagnostic symptoms associated with obsessive-compulsive disorder (OCD), anorexia nervosa, and bulimia nervosa [2,3,4,5,6,7,8,9]. When combining perfectionism with procrastination, the effects can be detrimental to many aspects of life, causing fear of failure and constant task delay, which, more often than not, leads to unsatisfactory results [10,11,12]. Yet, there may be a form of procrastination that has adaptive benefits and, instead of contributing to an obsession, works together with perfectionism to positively influence outcomes.

Procrastination is generally conceptualized based on a unidimensional model, focusing on the maladaptive aspects and consequences of procrastination [13,14,15]. Several multidimensional models for procrastination have been proposed in more recent years, attempting to evaluate the potential existence of not only a maladaptive (negative) form of procrastination, but also an adaptive (positive) form [16,17,18,19]. Presently, the association between perfectionism and procrastination remains inconsistent, with the concept of the existence of adaptive procrastination under heavy scrutiny [20,21]. We seek to develop a more cogent representation of the link between perfectionism and procrastination, as well as evaluate the potential existence of an adaptive form of procrastination. Our goal is to investigate the individual differences between maladaptive and adaptive forms of both perfectionism and procrastination while providing evidence of some links between the two. These individual components include conscientiousness, fear of failure, temporal orientation, the space-time metaphor, motivation and self-regulation, and emotion.

### 1.1. Perfectionism 

Research on perfectionism has made great strides throughout the 20th century in establishing the adaptability of this behavior. Over time, it has developed into a clear multidimensional model, with several different types. Hewitt and Flett [22] created a Multidimensional Perfectionism Scale (MPS) that splits perfectionism into three types: self-oriented, other-oriented, and socially prescribed. People are either focused on participating in a behavior for themselves or in an effort to please others or avoid social ridicule. These dimensions don’t make a distinction between adaptive or maladaptive types, but rather they differentiate between the motivations for perfectionistic behavior. Other multidimensional models are focused on classifying the behavior into groups. Frost et al. [1] classified perfectionism through several aspects of life including concern over mistakes, parental criticism, personal standards, parental expectations, doubts about actions, and organization. While these two multidimensional models of perfectionism seem to be measuring different aspects of perfectionism, Frost et al. [23] analyzed these two models and found two significant factor loadings. The first factor, which consisted of the categories of organization, personal standards, other-oriented perfectionism, and self-oriented perfectionism, seemed to represent the adaptive facets of perfectionism. The second factor consisted of parental criticisms, parental expectations, doubts about actions, concern over mistakes, and socially prescribed perfectionism, all of which seemingly reflected the maladaptive facets of perfectionism [23,24]. Further research contributed to this two-factor approach to perfectionism, providing supporting evidence that self-oriented perfectionism was associated with many adaptive facets of perfectionism, while socially-prescribed perfectionism, concern over mistakes, parental criticisms, and parental expectations were associated with the maladaptive facets of perfectionism [1,25,26,27,28,29,30,31,32,33]. 

In addition, Terry-Short et al. [34] also sought to derive a clearer distinction between adaptive and maladaptive perfectionism through the lens of learning theory. In their study, Terry-Short and colleagues [34] examined multiple populations that are known for their perfectionistic tendencies. Female athletes, clinically depressed female patients, and females suffering from eating disorders were compared to a control group. They found that athletes showed the highest levels of adaptive perfectionism, while women with eating disorders scored highest on maladaptive perfectionism [34]. The key to this differentiation between adaptive and maladaptive perfectionism was behavioral reinforcement. Athletes are encouraged toward personal achievement, which boosts self-esteem. In contrast, individuals with eating disorders are reinforced with images that remind them of what they are lacking. The Positive and Negative Perfectionism Scale (PNP) developed by Terry-Short et al. [34] has been adapted as a measure of multidimensional perfectionism across numerous areas of research, including procrastination, disordered eating, coping strategies, constructive and categorical thinking, satisfaction with life, cognitive dysfunction, neurocognition, self-evaluations, fatigue, body dissatisfaction, academic efficacy, dimensions of strain, vigor, and many aspects of negative affect [35,36,37,38,39,40,41,42,43,44,45,46,47]. 

For the purpose of this study, the instruments used to assess both positive and negative perfectionism in Terry-Short et al. [34] were used to represent adaptive and maladaptive perfectionism. Additionally, self-oriented perfectionism was used to illustrate adaptive perfectionism. Concern over mistakes and parental criticism were also used to represent maladaptive perfectionism, as well as to emphasize the connections with conscientiousness and fear of failure, respectively. Parental criticism was preferred over parental expectations due to previous research providing evidence of parental criticism being exclusively maladaptive perfectionism, whereas parental expectations have yet to be consistently identified as either adaptive or maladaptive perfectionism [29,31,32,33].

### 1.2. Procrastination

Procrastination is most commonly viewed as the purposeful delay of a task and is seemingly inevitable for college students. Ellis and Knaus [48] reported that 95% of college students have engaged in procrastination, while approximately 20% of adults claim to experience procrastination chronically [49]. The widespread use of procrastination and the problems it causes has prompted extensive research to be conducted to locate the source of this purposeful delay. The causes of procrastination have been found to range from task characteristics to individual characteristics, and even to demographics [13]. One identified source of this delay is procrastinators’ inability to manage their time correctly. They tend to be misguided in assigning time to complete a task, believing that the task ahead of them will not require much time and effort and, therefore, intentionally delaying the task [50]. Another source of this delay stems from task aversion. If a certain task is found to be unpleasant (or aversive), those who are attempting to complete the task may experience some difficulty in doing so. They tend to avoid tasks that are unpleasant in favor of those that are not [13].

The concept of procrastination currently exists in a unidimensional model, labeled as only existing in a maladaptive form. Its negative reputation stems from many researchers who have provided evidence that links procrastination to worse performances, poor health, and a decrease in well-being [51,52].

One of the most notable unidimensional models of procrastination was developed by Ferrari [15] who divided the concept of procrastination into three different types: avoidant, arousal, and decisional procrastination. Arousal procrastination is measured by the propensity to put off tasks in lieu of thrill-seeking while avoidance procrastination reflects a need for self-preservation [14]. Decisional procrastination, on the other hand, is “an inability to make decisions coupled with a sense of pessimism about reaching a satisfactory decision” [53] (pp. 838). This model of procrastination has since been analyzed by Steel [14], who determined that these three different modes of procrastination load onto only one factor, a universal factor of procrastination. Another pioneer in procrastination research and the development of an instrument to measure procrastination was Lay [54]. He investigated the impact that individual differences and situational aspects have on an individual’s propensity to procrastinate. From his research, he created the General Procrastination Scale (GPS), which focuses on the organization or disorganization displayed by procrastinators across contexts [54].

However, in recent years, a multidimensional model of procrastination was introduced by Chu and Choi [16]. They claimed that the concept of procrastination wasn’t as black and white as many had originally thought and proposed the existence of three distinct forms of procrastination: non-procrastinators, active (adaptive) procrastinators, and passive (maladaptive) procrastinators. Maladaptive procrastinators, they argued, are our “typical” procrastinators, those who put off completing a task due to a number of possible reasons, such as fear of failure or task aversion. They fail to appropriately manage their time and, therefore, are more likely to quit on difficult or anxiety-inducing tasks. On the other hand, adaptive procrastinators intentionally delay their tasks and prefer to work under a time pressure, which increases their motivation and enables them to satisfactorily complete their tasks before a deadline [16]. Support for adaptive procrastination illustrates an ability for an individual to procrastinate but still be high achieving. Ferrari et al. [55] found that students who attended very selective universities claimed to be procrastinators more often than students who attended nonselective schools. For some people, efficiency may increase when they are under a time crunch, otherwise, they find themselves distracted and exploring other ideas [52]. Other research has continued to test the existence of adaptive and maladaptive forms of procrastination, yielding results that support the differentiation between the two [18,56].

### 1.3. Variables Linking Perfectionism and Procrastination 

#### 1.3.1. Conscientiousness

The connection between conscientiousness and perfectionism has been extensively investigated and the results have been mostly universally agreed upon. Previous research has found that self-oriented perfectionism, most commonly associated with the adaptive aspects of perfectionism, was strongly associated with conscientiousness, while concern over mistakes, most commonly associated with the maladaptive aspects of perfectionism, was negatively associated with conscientiousness [26,57]. However, the link between procrastination and conscientiousness is still subject to debate despite a respectable amount of research on the two concepts. Using the multidimensional model of procrastination introduced by Chu and Choi [16], Choi and Moran [17] investigated the link between maladaptive procrastinators, adaptive procrastinators, and conscientiousness. From their analysis, they discovered that maladaptive procrastinators had a strong negative association with conscientiousness, while adaptive procrastinators had no association with conscientiousness [17]. This research contradicted a lot of previous research done on procrastination. Steel [13], one of the most notable researchers on procrastination and conscientiousness, demonstrated through a meta-analytic review how conceptual and literal procrastination is representative of low conscientiousness. He concluded that lack of impulse control, time management, persistence, and discipline were all inversely associated with conscientiousness and were representative of procrastination [13]. For the purpose of this study, we focused on the impulsivity and task aversiveness factors of procrastination that link to conscientiousness. These traits are in direct opposition to conscientiousness and, as Steel [13] asserts, are fundamental to procrastination. Since impulsivity and task aversion seem to conceptually relate to Ferrari’s [15] arousal and avoidant procrastination, we decided to investigate any correlation these two forms of procrastination had with conscientiousness.

**Hypothesis** **1:** *Adaptive procrastination is positively associated with conscientiousness, which creates a link between both adaptive aspects of procrastination and perfectionism, while the specific aspects of maladaptive procrastination, arousal, and avoidant procrastination, are negatively associated with conscientiousness.*

#### 1.3.2. Emotion (Depression, Stress, Anxiety, and Satisfaction with Life)

Emotion is an essential factor to consider when investigating the link between procrastination and perfectionism. The dual-process model, originally proposed to be adaptable to perfectionism by Slade and Owens [58], was further investigated by Bergman, Nyland, and Burns [39]. The dual-process model differentiates between an adaptive and a maladaptive mode of perfectionism on a functional basis. The underlying functional processes that lend to this distinctive difference include emotional states and cognitive processes. Bergman, Nyland, and Burns [39] found that maladaptive perfectionism is related to many negative characteristics of emotion, such as heightened levels of depression, anxiety, and stress, along with a decreased satisfaction with life and a negative view of the future due to rumination over potential future failures.

Procrastinators have been found to exhibit many of the characteristics associated with maladaptive perfectionism in this dual process model [59,60,61]. However, past research has linked procrastination and maladaptive aspects of perfectionism based on a unidimensional model of procrastination. We will attempt to adapt the dual-process model to procrastination the same way it was successfully applied to perfectionism.

**Hypothesis** **2:** *A dual-process model, representative of emotional aspects, can be applied to the construct of procrastination, just as it has been applied to perfectionism, therefore linking the two.*

#### 1.3.3. Fear of Failure

Procrastinators have been frequently linked to the maladaptive aspects of perfectionism, most notably to the excessive fear of failure, placing unrealistic demands on themselves, and the endorsement of irrational beliefs [10,12,62,63]. For the purpose of this study, fear of failure will be the focus of the link between the maladaptive subsets of procrastination and perfectionism.

A multidimensional model for fear of failure has been recently created by Conroy, Willow, and Metzler [64], identifying five individual aspects of fear of failure: experiencing shame and embarrassment, devaluing one’s self-esteem, having an uncertain future, important others losing interest, and upsetting important others. Several studies have linked all five fear of failure aspects to both parental expectations and parental criticisms [65,66].

However, when investigating the adaptability of the Multidimensional Perfectionism Scale (MPS) to sporting contexts, Anshel and Eom [67] and Dunn, Dunn, and Syrotuik [68] identified four dimensions from their analyses: personal standards, concern over mistakes, parental criticism (or perceived parental pressure), and coach criticism (or perceived coach pressure). Perceived parental pressure, which combines aspects of parental expectations and parental criticisms, failed to correlate with all five dimensions of fear of failure, yielding significant correlations with only two of the dimensions [69]. Given the findings of Sagar and Stoeber [69], as well as the evidence previously discussed regarding parental expectations not always demonstrating maladaptive facets of perfectionism, we intend to utilize the parental criticism scale as a potential measure of fear of failure in the present study.

In addition to perfectionism and procrastination, fear of failure has an established link to motivation, a key component of procrastination [11]. A study conducted by Kubanek, Snyder, and Abrams [70] found that students were more motivated to obtain better grades at the risk of “losing” something rather than the prospect of “gaining” a reward for their behavior. Students feared the consequences of failure more than they sought a reward for completing a task, thereby effecting their motivational stimulus. However, past research using fear of failure as the link between these two concepts has used the unidimensional model of procrastination. We will analyze this relationship through the lens of the multidimensional model of procrastination, which includes examining potential links between fear of failure and adaptive procrastination.

**Hypothesis** **3:** *Procrastination will reflect the link between fear of failure and perfectionism, meaning adaptive procrastination will not relate to fear of failure, but maladaptive procrastination will.*

#### 1.3.4. Temporal Orientation

At present, there is some evidence that procrastination and perfectionism have a relationship on a behavioral and cognitive basis. Further analysis of this idea reveals a need to examine the concept of temporal orientation. The idea of “orientation” in temporal awareness was heavily discussed by Philip Zimbardo in his development of the Zimbardo Time Perspective Instrument (ZTPI). The ZTPI classifies an individual’s relationship with time into past, present, and future orientations, as well as further delineating them into fatalistic and hedonistic outlooks, creating a five-factor model [71]. These time perspectives provide a glimpse into an individual’s personality since an individual’s temporal awareness can be heavily influenced by one’s decision making processes, judgments, and actions, as well as an individual’s appraisals of personal experiences [72,73].

The first perspective, past-negative, represents an aversive view of the past, causing negative emotions such as pain, trauma, and regret. It has strong negative associations with consideration of future consequences, impulse control, and self-esteem, as well as strong positive associations with depression, trait anxiety, and negative affect [71,74]. Due to these associations, the past-negative perspective seemingly has a relationship with maladaptive procrastination, which is characterized by many of these variables.

Another perspective that exists in direct opposition to the past-negative perspective is the past-positive perspective. This perspective relates to a happy and nostalgic view of the past. It has shown to have strong negative associations with trait anxiety and depression, along with a strong positive association with self-esteem and subjective well-being [71,75]. These associations relate to adaptive perfectionism and have the potential to relate to adaptive procrastination as well.

The third perspective, future orientation, is dominated by a focus on future goals and potential rewards. Strong negative associations with depression and trait anxiety, and strong positive associations with conscientiousness, consideration of future consequences, impulse control, and subjective well-being have been found [71,75]. Similar to the past-positive perspective, many of these associations relate to adaptive perfectionism and potentially adaptive procrastination.

The present-hedonistic perspective is closely related to impulsivity. It represents risk-taking behavior and does not take into account future consequences that may result from their actions. Further research has supported this view that present-oriented individuals engage in risky behaviors, such as driving under the influence [76]. This perspective has revealed strong negative associations with conscientiousness, impulse control, and consideration of future consequences, and a strong positive association to depression, all of which seem to relate to maladaptive procrastination [71].

Lastly, the present-fatalistic perspective is reflective of hopeless views of the future and having no control over future events. They do not have a healthy or happy outlook on life. It has shown to have strong negative associations with conscientiousness, consideration of future consequences, impulse control, and self-esteem, and strong positive associations to trait anxiety, depression, and negative affect [71,74]. As with the present-hedonistic perspective, the present-fatalistic perspective objectively has a relationship with maladaptive procrastination.

Overall, three classifications were made for the five perspectives of the ZTPI. The two perspectives considered the least adaptive in regard to predicting psychological adjustment were the past-negative and present-fatalistic perspectives. The future-oriented and past-positive perspectives were determined to be the most adaptive perspectives, and the present-hedonistic perspective was ambiguous in its ability to predict either adaptive or maladaptive psychological adjustment [71,72]. With all five perspectives of the ZTPI considered, we hope to establish the relationships between each perspective and the multidimensional models of both perfectionism and procrastination.

**Hypothesis** **4:** *Maladaptive procrastination will correlate with the past-negative, present-hedonistic, and present-fatalistic perspectives of the ZTPI. Adaptive procrastination, on the other hand, will correlate with the past-positive and future orientation perspectives, creating a relation to adaptive perfectionism.*

#### 1.3.5. Motivation and Self-Regulation

Motivation and self-regulation are two concepts that are frequently conceptually intertwined, often used in tandem when analyzing certain constructs, such as procrastination. Self-regulation has been defined as “any process by which an organism regulates its state, encompassing all manners of goal pursuit [77],” [78] (p. 5) and motivation has been defined as “the reasons why people pursue their goals” [78] (p. 7). Since an individual’s ability to regulate oneself and their reasons for pursuing a specific goal play an important role in both perfectionism and procrastination, we will attempt to utilize various methods of examination that relate to both motivation and self-regulation to support the concept of adaptive procrastination.

##### Boredom Proneness

Proneness to boredom is a factor that has been linked to procrastination, as well as motivation. Blunt and Pychyl [53] investigated the relationship between the different types of procrastination proposed by Ferrari [15] and proneness to boredom. They found a positive association between decisional and arousal procrastination and proneness to boredom [53]. However, further research has shown that boredom proneness is not a unidimensional concept. Vodanovich, Wallace, and Kass [79] conducted research on boredom proneness and connected the concept with stimulation. Delaying a task because of boredom may not necessarily mean that the individual doubts their abilities to complete a task. In fact, the exact opposite may be true. The measurements of boredom reveal two distinct sources of motivation, similar to the other constructs in this study. This multidimensionality shows that internal, as well as external, sources of motivation can be linked to boredom. A lack of internal stimulation reveals an inability to properly motivate oneself to begin a task, while external stimulation relates a need for constant change and variety in life [79]. We will consider the relationship between these two types of stimulation and the multidimensional models of perfectionism and procrastination.

**Hypothesis** **5:** *Both adaptive procrastination and adaptive perfectionism will associate with internal stimulation, but not with external stimulation. Maladaptive procrastination, more specifically arousal procrastination, will associate with external stimulation, while avoidant procrastination will negatively associate with internal stimulation.*

##### Need for Affect

Another variable in relation to motivation stems from a need for affect (NFA). People are intrinsically motivated to either approach or avoid an emotion-inducing event that could cause emotions ranging from anxiety to self-satisfaction [80,81]. In relation to procrastination, NFA can persuade an individual to either approach or avoid a task purely based on the emotion-inducing consequences. We will investigate this link between the two types of NFA and its potential associations to perfectionism and procrastination.

**Hypothesis** **6:** *Adaptive procrastination and perfectionism will link to NFA approach, while maladaptive procrastination and perfectionism, more specifically, avoidant procrastination will link to NFA avoidance.*

##### Self-Efficacy and Euthymia 

The relationship between procrastination and motivation is also evident through studies related to self-efficacy. Tuckman and Sexton [82] conducted a study in which individuals were presented a task to compete in a limited amount of time. Participants that believed that they would be able to accomplish the task (high self-efficacy) performed better than they had expected while those that doubted their abilities (low self-efficacy) did worse than anticipated [82]. This provides further evidence that there is a strong relationship between determination and accomplishment. For example, people tend to have an unusual desire to clean their houses when they should be studying or working. It is not an enjoyable task any other time but it results in a momentary sense of accomplishment and a positive mood.

Further support for the importance of self-regulation is illustrated through euthymia. The euthymia scale examines the idea of high self-esteem and satisfaction with self in defining the relationship between constructs. The clinical concept of euthymia evaluates positive mental health. A state of euthymia includes a positive emotional state, resiliency after conflict, and a lack of mood disturbances [83]. The positive emotional response elicited through euthymia can be directed toward a task and, therefore, increase motivation. It is possible that this increase in motivation and self-efficacy has an influence on the procrastination of an individual, and we seek to analyze whether adaptive procrastinators have the motivational and self-efficacy effects that are observed in adaptive perfectionists.

**Hypothesis** **7:** *Euthymia and self-efficacy will correlate with adaptive forms of both procrastination and perfectionism.*

#### 1.3.6. Time Management

Adaptive perfectionists have better control over anxiety due to internal influences such as conscientiousness and motivation. Individuals that are highly intrinsically motivated may be perfectionists but are unlikely to be chronic procrastinators because they are able to manage their time in an organized fashion [84]. Procrastinators have been proven to be the opposite of adaptive perfectionists, intentionally delaying tasks and causing time management issues [50,85,86]. However, as Chu and Choi [16] assert using their multidimensional model of procrastination, adaptive procrastinators intentionally create a time pressure for themselves, therefore managing their time in a way that best suits their preferences. Adaptive procrastinators adequately manage their time to their liking just as adaptive perfectionists do to accomplish a task.

Another way to understand an individual’s relationship with time can be seen through the space-time metaphor. This metaphor examines the relationship between ideas about the “self” in relation to ideas about time and consists of two metaphors: The moving time metaphor and the moving ego metaphor. The moving time metaphor consists of a stationary observer who believes they have no power over time and events that will or have come to pass. The moving ego metaphor involves an active sense of agency, revolving around an ego that actively moves across time [87,88]. The time question, which is phrased as an event occurring on Wednesday has been moved back two days, is a common way to assess the space-time metaphor. Those who exhibit the moving time metaphor are expected to interpret the time question as moving “back” to Friday, whereas those demonstrating the moving ego metaphor should interpret the question as moving “back” to Monday [89]. Researchers conducted a study using this idea and found that an individual’s answer to a question about a moving deadline mirrored self-efficacy. Stronger emotional responses to a change in deadline were associated with a higher sense of agency [87]. We will examine the possible link between the space-time metaphor and both procrastination and perfectionism.

**Hypothesis** **8:** *Adaptive procrastinators and adaptive perfectionists will associate with the ability to manage time appropriately and demonstrating the moving ego metaphor.*

The main objective of this research was to understand the relationship between the two constructs of perfectionism and procrastination through the lens of conscientiousness, emotion, motivation and self-regulation, fear of failure, the space-time metaphor, and temporal orientation. It will be important to determine whether their expression is more alike than different through a new model of this relationship. Various measures were used to assess this relationship, including subscales of the Frost Multidimensional Perfectionism Scale (FMPS), multiple measures of procrastination such as the General Procrastination Scale (GPS) and the Consequences of Procrastination Scale (COPS), a short-form of conscientiousness, NFA, euthymia, boredom proneness, depression, anxiety, stress, satisfaction with life, the space-time metaphor, and temporal orientation. Throughout this study, researchers sought to confirm the concept of adaptive procrastination, clarify the nature of its relationship with perfectionism, and bring a new perspective to procrastination in academia.

## 2. Materials and Methods

### 2.1. Participants

The participants in this study consisted of 206 introductory level psychology students at Grand Valley State University. This was a convenience sample as students participated on a voluntary basis and received points for their participation. The sample was 57.2% female. The ethnic demographics were consistent with the university’s incoming class of 2015–2016. We estimate that 82.6% of participants were Caucasian-American, 5.2% African American, 4.8% Hispanic-American, 2% Asian-American, and 0.4% Native American. The age range of participants was from 18–29.

### 2.2. Procedure

Participants were distributed survey materials through their Introductory Psychology class. Informed consent was obtained from potential participants prior to the questionnaire. The questionnaire included 236 questions and each participant answered them in randomized order. Upon completion, participants were debriefed about the survey, compensated with one class credit hour, and thanked for their participation. Results from the questionnaires were then organized and analyzed using SAS software, Version 9.4 of the SAS System for Unix. Copyright © 2020 SAS Institute Inc. SAS and all other SAS Institute Inc. product or service names are registered trademarks or trademarks of SAS Institute Inc., Cary, NC, USA.

### 2.3. Measures

Perfectionism, procrastination, fear of failure, conscientiousness, temporal orientation, anxiety, euthymia, depression, boredom proneness, need for affect, satisfaction with life, stress, and the space-time metaphor were operationalized on the questionnaire through a combination of the following scales:

#### 2.3.1. Positive and Negative Perfectionism Scale (PNP) 

The PNP consists of two subscales measuring positive/adaptive (PP) and negative/maladaptive procrastination (NP) separately [34]. It contains 40 items that must be measured on a Likert scale of 1 (strongly disagree) to 5 (strongly agree). Scores were obtained by averaging the set of items that represent adaptive perfectionism against the scores for maladaptive perfectionism. Sample items include “I gain deep satisfaction when I have perfected something” and “Other people expect nothing less than perfection from me.” It was our intention to use the short form of the PNP, which consisted of 20 items, but a few items were accidentally omitted. The Cronbach’s alphas for the PP and NP scales were 0.65 and 0.80 respectively and, while lower than ideal, the scales were retained. 

#### 2.3.2. Multidimensional Perfectionism Scale (MPS)

The MPS contains three subscales measuring three subsets of perfectionism that are applied across the self and social contexts: self-oriented perfectionism (SOP), other-oriented perfectionism (OOP), and socially prescribed perfectionism (SPP) [90]. For the purpose of this study, only the SOP subscale, consisting of 15 items, was included to operationalize adaptive perfectionism. Sample items include “I am perfectionistic in setting my goals” and “It makes me uneasy to see an error in my work.” Items are measured on a 7-point Likert scale, ranging from 1 (strongly disagree) to 7 (strongly agree). The Cronbach’s alpha for the SOP was 0.80.

#### 2.3.3. Frost Multidimensional Scale (FMPS) 

The FMPS is a perfectionism scale that categorizes perfectionism into aspects of life including concern over mistakes (CM), parental criticism (PC), personal standards (PS), parental expectations (PE), doubts about actions (DA), and organization (O) [1]. This study only included items from the CM (“If I fail partly, it is as bad as being a complete failure”) and PC (“My parents never tried to understand my mistakes”) subscales in order to adequately measure conscientiousness and fear of failure. There are nine CM items and four PC items that are measured on a Likert scale of 1 (strongly agree) to 5 (strongly disagree). The Cronbach’s alphas for the CM and PC were 0.90 and 0.83, respectively. 

#### 2.3.4. Consequences of Procrastination Scale (COPS-10)

The COPS 10-item perfectionism scale was adapted to measure the consequences of procrastination rather than perfectionism [91]. The 10 items were formatted to measure the presence of adaptive procrastination. Sample items include “Being a procrastinator pushes me to stay on track in my performances” and “Being a procrastinator gets me to decrease my productivity”. Participants responded to questions on a Likert scale of 1 (extremely untrue of me) to 5 (extremely true of me). The Cronbach’s alpha for the adapted COPS-10 was 0.91. 

#### 2.3.5. General Procrastination Scale (GPS)

The GPS measures the individual’s level of self-management on a scale from neurotic disorganization to organization [54]. This scale consists of 20 items in total, all measured on a Likert scale of 1 (extremely uncharacteristic) to 5 (extremely characteristic). Half of the items on the scale are reverse-scored to measure neurotic disorganization. The Cronbach’s alpha for the GPS was 0.82. 

#### 2.3.6. Pure Procrastination Scale (PPS)

The PPS is a 12-item scale that measures procrastination in three different types: arousal (“I don’t get things done on time”), avoidant (“I don’t make decisions unless I really have to”), and decisional (“Even after I make a decision, I delay acting upon it”) [13,14]. Arousal procrastination (ARO) is measured by the propensity to delay a task for immediate gratification while avoidance procrastination (AVO) reflects a need for self-preservation, and decisional procrastination (DEC) refers to a delay in decision-making. The Cronbach’s alphas for the DEC, AVO, and ARO were 0.89, 0.81, and 0.79, respectively. 

#### 2.3.7. The NEO Five Factor Inventory: Form S (NEO-FFI)

The NEO-FFI is a 60-item short-form version of the NEO Personality Inventory [92]. It contains 12 items each for the 5 factors: Neuroticism (N), Extraversion (E), Openness (O), Agreeableness (A), and Conscientiousness (C). We used the conscientiousness scale to measure an individual’s level of conscientiousness. Items are measured on a Likert scale of 1 (strongly disagree) to 5 (strongly agree). The Cronbach’s alpha for the NEO-FFI was 0.82. 

#### 2.3.8. The Perceived Stress Scale (PSS)

The PSS is a 14-item scale that measures individual stress level through how an individual rates their life events as having been stressful throughout the last month, as well as demographics [93]. Items are measured on a Likert scale of 0 (never) to 4 (very often). The Cronbach’s alpha for the PSS was 0.84. 

#### 2.3.9. The Center for Epidemiological Studies Depression Scale (CES-D)

The CES-D short form is an 11-item scale that measures depressive symptoms with four factors [94]. These factors include positive affect, depressed affect, interpersonal problems, and somatic complaints. Using a 3-point Likert scale, ranging from 1 (hardly ever or never) to 3 (much or most of the time), respondents indicate how they felt or behaved in the last week. The Cronbach’s alpha for the CES-D short-form was 0.84. 

#### 2.3.10. State-Trait Anxiety Inventory (STAI-SF)

This study uses a shortened version of the STAI-SF, which consists of six items to measure dispositional anxiety and individual differences [95]. All items are measured on a Likert scale of 0 (not at all) to 3 (very much so). The Cronbach’s alpha for the STAI-SF was 0.83. 

#### 2.3.11. The Extended Satisfaction with Life Scale (ESWLS)

The ESWLS is a scale designed to measure life satisfaction in different areas of life (social, sexual, general, etc.) [96]. The general satisfaction with life subscale, consisting of six items, is the only subscale used in the present study. It is measured on a 5-point Likert scale. The Cronbach’s alpha for the ESWLS was 0.88. 

#### 2.3.12. Zimbardo Time Perspective Inventory (ZTPI)

The ZTPI measures temporal orientation from past, present, and future perspectives [71]. This scale consists of 52 items. All items are measured on a Likert scale of 1 (very uncharacteristic) to 5 (very characteristic). Multiple items were reverse-scored to measure fatalistic and hedonistic perspectives of time. Past-oriented individuals dwell on mistakes of the past, and this may either lead to a desire for perfection in the future or an inability to begin a task for fear of failure. People that are present-oriented seek quick pleasure with little concern for consequences. Cronbach’s alphas for the past-negative (PN) (2 items were dropped to improve the scale’s alpha), present-hedonistic (PH), future orientation (FUT), past-positive (PP), and present-fatalistic (PF) were 0.73, 0.84, 0.78, 0.81, and 0.76 respectively.

#### 2.3.13. Boredom Proneness Scale-Short Form (BPS-SF)

The Boredom Proneness Scale measures motivation through external stimulation and internal stimulation [79]. A lack of internal stimulation reveals an inability to properly motivate oneself to begin a task, while external stimulation relates to a need for constant change and variety in life. The BPS consists of six internal stimulation items (“I find it easy to entertain myself”) and six external stimulation items (“Many things I have to do are repetitive or monotonous”), all measured on a Likert scale of 1 (strongly agree) to 7 (strongly disagree). The Cronbach’s alphas for the internal stimulation and external stimulation scales were 0.73 and 0.67, respectively. 

#### 2.3.14. Need for Affect Questionnaire-Short Form (NAQ-S)

The NAQ-S is a 10-item scale consisting of five items related to the approach subscale (“I feel that I need to experience strong emotions regularly”) and five items related to the avoid subscale (“I would prefer not to experience either the lows or highs of emotion”) [80]. All items are measured on a 7-point Likert scale, ranging from −3 (strongly disagree) to 3 (strongly agree). The avoid subscale items are reverse scored. The Cronbach’s alphas for the NAQ-Avoid and the NAQ-Approach were 0.79 and 0.81, respectively. 

#### 2.3.15. Euthymia 

Euthymia is measured by 10 true-false statements that determine whether participants have a positive outlook on life through questions about their overall mental and physical health [83]. Sample items include “I am able to adapt to changing situations”, and “I generally feel cheerful and in good spirits”. Items on this scale are scored by assigning a “0” for false statements and a “1” for true statements, with 10 being the highest score possible. One item was dropped to improve the Cronbach’s alpha, which for the Euthymia scale was 0.72.

#### 2.3.16. Space-Time Metaphor

The space-time metaphor measures personality and temporal awareness through the metaphors of moving time and moving ego [87]. In the moving ego metaphor, time is conceived as a backdrop for which the “self” moves across and the moving time metaphor, time is conceived as the motion, while the “self” is stationary. This idea is measured through responses to a single statement about a moving deadline. Participants who answer Monday are assigned a score of “1” to represent the moving ego metaphor and participants who answer Friday are assigned a score of “2” to represent the moving time metaphor. The Space-Time Metaphor is a single-item scale. 

Table 1 Descriptive statistics, Cronbach’s alphas, and correlations between adaptive and maladaptive perfectionism, adaptive and maladaptive procrastination, emotion, fear of failure, conscientiousness, motivation and self-regulation, the space-time metaphor, and temporal orientation.

## 3. Results

All means, standard deviations, correlations, and Cronbach’s alphas are reported in Table 1.

### 3.1. Conscientiousness

It was hypothesized that adaptive procrastinators and adaptive perfectionists would demonstrate a positive association with conscientiousness. The results were only partially consistent with our predictions; positive perfectionism correlated positively with conscientiousness (r = 0.44, *p* < 0.001), as well as self-oriented perfectionism (r = 0.45, *p* < 0.001). However, our adaptive procrastination scale, the consequences of procrastination scale, had no association with conscientiousness (r = 0.04, *p* = NS). It was also expected that arousal and avoidant procrastination, both considered maladaptive forms of procrastination, would negatively associate with conscientiousness due to their relation to impulsivity and task aversion, respectively. The results supported our hypothesis; conscientiousness correlated negatively with both arousal procrastination (r = −0.57, *p* < 0.001) and avoidant procrastination (r = −0.58, *p* < 0.001).

### 3.2. Emotion (Depression, Stress, Anxiety, and Satisfaction with Life)

It was thought that the concept of procrastination could be applicable to the dual process model, just as perfectionism had been applied to this model. The data collected from this experiment only partially supports this hypothesis; the consequences of procrastination scale had no association with stress (r = −0.12, *p* = NS), depression (r = −0.03, *p* = NS), anxiety (r = −0.06, *p* = NS), or satisfaction with life (r = 0.06, *p* = NS). The adaptive perfectionism variable positive perfectionism correlated positively with satisfaction with life (r = 0.30, *p* < 0.001), correlated negatively with depression (r = −0.16, *p* < 0.05) and anxiety (r = −0.19, *p* < 0.01), but had no association with stress (r = −0.12, *p* = NS). The other adaptive perfectionism variable self-oriented perfectionism only positively correlated with satisfaction with life (r = 0.17, *p* < 0.05), and had no association with stress (r = 0.13, *p* = NS), depression (r = 0.03, *p* = NS), and anxiety (r = 0.02, *p* = NS).

On the other hand, our maladaptive procrastination variable demonstrated some significant correlations; the general procrastination scale had positive correlations with stress (r = 0.19, *p* < 0.01) and depression (r = 0.15, *p* < 0.05), a negative correlation with satisfaction with life (r = −0.23, *p* < 0.01), but had no association with anxiety (r = 0.12, *p* = NS). 

All measures of maladaptive perfectionism (concern over mistakes, parental criticism, and negative perfectionism) demonstrated similar correlations as seen with maladaptive procrastination; concern over mistakes positively correlated with stress (r = 0.42, *p* < 0.001), depression (r = 0.36, *p* < 0.001), and anxiety (r = 0.32, *p* < 0.001), while negatively correlated with satisfaction with life (r = −0.30, *p* < 0.001). Parental criticism also had a positive correlation with stress (r = 0.39, *p* < 0.001), depression (r = 0.44, *p* < 0.001), and anxiety (r = 0.28, *p* < 0.001), as well as a negative correlation with satisfaction with life (r = −0.44, *p* < 0.001). Furthermore, negative perfectionism correlated positively with stress (r = 0.59, *p* < 0.001), depression (r = 0.42, *p* < 0.001), and anxiety (r = 0.32, *p* < 0.001), while negatively correlated with satisfaction with life (r = −0.28, *p* < 0.001).

### 3.3. Fear of Failure

Maladaptive procrastinators were expected to have a negative association to fear of failure, similar to that of maladaptive perfectionism. This hypothesis was partially supported by the data collected; fear of failure had positive correlations with maladaptive perfectionism variables negative perfectionism (r = 0.30, *p* < 0.001) and concern over mistakes (r = 0.47, *p* < 0.001), as well as a negative correlation with positive perfectionism (r = −0.25, *p* < 0.001), but had no association with self-oriented perfectionism (r = −0.04, *p* = NS). In contrast, the consequences of procrastination scale had no association with fear of failure (r = −0.05, *p* = NS) while the general procrastination scale demonstrated a positive correlation with fear of failure (r = 0.19, *p* < 0.01).

### 3.4. Temporal Orientation

It was hypothesized that adaptive procrastinators would mimic adaptive perfectionism’s associations with positive conceptions of time, such as the past-positive and future-oriented perspectives. Our data only partially supports this hypothesis; the consequences of procrastination scale had no association with past-positive (r = 0.03, *p* = NS) or future orientation (r = 0.01, *p* = NS). Yet, positive perfectionism had a positive correlation with future orientation (r = 0.37, *p* < 0.001) and past-positive (r = 0.36, *p* < 0.001), but self-oriented perfectionism only had a positive correlation with view is future-oriented (r = 0.53, *p* < 0.001) and had no association with view of past as positive (r = 0.09, *p* = NS).

It was also hypothesized that maladaptive procrastination would associate with the negative perspectives of time. This hypothesis was supported by our data; the general procrastination scale demonstrated the expected correlations with the negative perspectives of time. The general procrastination scale had a positive correlation with past-negative (r = 0.28, *p* < 0.001), present-fatalistic (r = 0.21, *p* < 0.01), and present-hedonistic (r = 0.17, *p* < 0.05), as well as a negative correlation with future orientation (r = −0.61, *p* < 0.001).

### 3.5. Boredom Proneness

Adaptive procrastinators and perfectionists were thought to have a positive association with internal stimulation, as well as no association with external stimulation, and our data only partially supports this hypothesis; the consequences of procrastination scale had no association with internal (r = 0.10, *p* = NS) or external stimulation (r = −0.04, *p* = NS). However, positive perfectionism had a positive correlation with internal stimulation (r = 0.35, *p* < 0.001) and a negative correlation with external stimulation (r = −0.17, *p* < 0.05), but self-oriented perfectionism had only a positive correlation with internal stimulation (r = 0.28, *p* < 0.001) and had no association with external stimulation (r = −0.05, *p* = NS).

It was also thought that maladaptive procrastinators who engaged in arousal procrastination would associate with external stimulation, and those who engaged in avoidant procrastination would associate with a lack of internal stimulation. The results were only partially consistent with our hypothesis; external stimulation had a positive correlation with both arousal procrastination (r = 0.20, *p* < 0.01) and avoidant procrastination (r = 0.17, *p* < 0.05). Yet, internal stimulation had a negative correlation with avoidant procrastination (r = −0.16, *p* < 0.05), but had no association with arousal procrastination (r = −0.14, *p* = NS).

### 3.6. Need for Affect

It was predicted that adaptive procrastinators and perfectionists were more likely to engage in an NFA approach. The results only partially supported this prediction; the NFA approach had a positive correlation with both positive perfectionism (r = 0.16, *p* < 0.05) and self-oriented perfectionism (r = 0.15, *p* < 0.05). However, NFA avoidance had no association with either positive perfectionism (r = 0.14, *p* = NS) nor self-oriented perfectionism (r = −0.03, *p* = NS). On the other hand, the consequences of procrastination scale had a positive correlation with NFA avoidance (r = 0.18, *p* < 0.01), but had no association with NFA approach (r = 0.03, *p* = NS).

In addition, maladaptive procrastination and perfectionism, more specifically avoidant procrastination, were predicted to positively associate with NFA avoidance. Our data did not support this prediction; NFA avoidance had a negative correlation with all maladaptive perfectionism variables: parental criticism (r = −0.16, *p* < 0.05), concern over mistakes (r = −0.26, *p* < 0.001), and negative perfectionism (r = −0.24, *p* < 0.001). However, NFA avoidance had no association with the general procrastination scale (r = −0.02, *p* = NS), as well as no association with avoidant procrastination (r = −0.13, *p* = NS).

### 3.7. Self-Efficacy and Euthymia

Adaptive procrastination and perfectionism were expected to exhibit euthymia and self-efficacy. Our hypothesis was supported by the results; the consequences of procrastination scale had a positive correlation with euthymia and self-efficacy (r = 0.15, *p* < 0.05). Positive perfectionism also demonstrated a positive correlation with euthymia and self-efficacy (r = 0.25, *p* < 0.001), but self-oriented perfectionism had no association with euthymia and self-efficacy (r = 0.05, *p* = NS).

### 3.8. Time Management

It was hypothesized that adaptive procrastinators would be similar to adaptive perfectionists in that they both would associate with the ability to manage time appropriately and relate to the moving ego metaphor. The results did not support this hypothesis; the ability to manage time appropriately had no association with either the consequences of procrastination scale (r = 0.07, *p* = NS) nor both adaptive perfectionism variable positive perfectionism (r = 0.09, *p* = NS) and self-oriented perfectionism (r = 0.06, *p* = NS).

However, some of the specific facets of procrastination that were assessed in our study had a positive correlation with the moving time metaphor; the moving time metaphor had a positive correlation with both decisional (r = 0.16, *p* < 0.05) and avoidant procrastination (r = 0.20, *p* < 0.01), but not arousal procrastination (r = 0.13, *p* = NS).

## 4. Discussion

Overall, we did not find evidence for the existence of an adaptive form of procrastination. However, the multidimensional model of perfectionism, as well as maladaptive procrastination, displayed many significant correlations, providing support for previous research and outlining the emergence of new relationships.

### 4.1. Conscientiousness

The data from our study replicated the results of previous research that found adaptive perfectionism positively correlated with conscientiousness and adaptive procrastination had no association with conscientiousness [17,26,57,97,98,99]. In addition, both arousal and avoidant procrastination correlated negatively with conscientiousness, supporting our hypothesis that these two specific aspects of procrastination relate to the concepts of impulsivity and task aversion.

### 4.2. Emotion (Depression, Stress, Anxiety, and Satisfaction with Life)

The results from the current study replicated some of the previous findings of Bergman, Nyland, and Burns [39]. Similar to Bergman, Nyland, and Burns [39], the present study found evidence of maladaptive perfectionism strongly relating to maladaptive characteristics, including an increase in stress, depression, anxiety, while demonstrating a decrease in satisfaction with life. In addition, the present study found that maladaptive procrastination was also related to maladaptive characteristics, providing further support for the evidence that maladaptive procrastination can be linked to maladaptive perfectionism through these characteristics [59,60,61].

However, adaptive perfectionism did not completely fit into the dual-process model described by Bergman, Nyland, and Burns [39]. While adaptive perfectionism was associated with an increase in satisfaction with life, as well as a decrease in both depression and anxiety, the present study revealed that adaptive perfectionism had no association with stress. One potential explanation for the lack of a significant negative correlation between stress and adaptive perfectionism is the diathesis-stress model. This model states that an individual may have some weakness or predisposition to a certain disease or psychopathology that can be triggered by specific stressful life events the individual experiences [100]. After this activation, the disorder can express itself in varying levels of severity [100]. While the diathesis was originally thought to be due to external or biological origins, further research has revealed the potential existence of diatheses based on personality and cognition [100,101]. Research conducted by Monroe and Simons [102] and Hammen [103] found that not only can personality and cognitive diatheses directly influence stress, but, more specifically, maladaptive characteristics of personality and cognition can be responsible for the creation of stressful life events [100].

The diathesis-stress model has been further investigated to the potential application to Hewitt and Flett’s [22] model of multidimensional perfectionism, which includes self-oriented, other-oriented, and socially-prescribed perfectionism. Hewitt and Flett [104] theorized that self-oriented perfectionists, while using what is often considered the adaptive form of perfectionism, would still experience maladaptive aspects of perfectionism, given a certain situation. They hypothesized that self-oriented perfectionists would fall victim to the maladaptive characteristics of perfectionism when exposed to achievement-related stressors due to their personality diathesis [104]. If a self-oriented perfectionist were to fail at a specific achievement-oriented goal, their self-esteem would falter, leaving them vulnerable to the emotional maladjustment. Others built onto this hypothesis, agreeing that adaptive perfectionists are more vulnerable to psychological distress when they do not achieve a personal goal since they lose their motivation to attain a perfect outcome [105,106,107]. Lee [100] further investigated this concept and discovered that both self-oriented and socially-prescribed perfectionism were associated with appraised achievement stress, but only self-oriented perfectionism was able to predict appraised achievement stress when controlling for general negative affect. These findings supported Hewitt and Flett’s [104] hypothesis that, when exposed to achievement-related stressors, self-oriented perfectionists may experience more maladjusted emotions than socially-prescribed perfectionists due to the nature of the event and the appraisal of the event as stressful.

Furthermore, additional research has found a difference between an individual’s perception of a stressful event and the objective stressful event [100]. Two individuals could have substantially different appraisals of a stressful event, with one individual viewing the event as an average stressor, while the other views the event on a much larger scale [100].

When applied to the multidimensional model of perfectionism, one could argue that adaptive perfectionists, while still experiencing the positive aspects of perfectionism, may be more susceptible to life stressors than non-perfectionists. The negative consequences associated with perfectionism, such as stress, may not be as readily seen due to the existence of these maladaptive emotions being dependent on life stressors and appraisals.

In addition, due to our sample population consisting exclusively of college students, the results from the current study may be representative of a particularly vulnerable population. Today, college students are experiencing stressors at an unprecedented level. The costs for tuition, room, and board are constantly on the rise, causing extreme levels of financial strain on college students who are already experiencing multiple serious stressors resulting from their enrollment in a university, such as stressors related to transitions, academics, expectations, the environment, diversity, and lack of resources [108]. Furthermore, the potential debt burden resulting from these college-related expenses adds another layer of complexity to the financial stressors college students experience [108]. Due to these detrimental future financial burdens that college students face, they may have to work part-time, and some full-time, in order to stave off financial ruin, adding yet another aspect of stress to a college student’s experience [109,110]. The effects of the stressors that college students face can be detrimental to their personal well-being, leading to higher levels of psychological distress, often characterized by low academic self-efficacy, high test anxiety, and an overall negative impact on academic performance [111]. Considering the number of stressors experienced by college students, and the added impact of a major life transition from home to university, the lack of association between stress and adaptive perfectionism found in a student population could be better understood [112].

### 4.3. Fear of Failure

While our measure of adaptive procrastination, the consequences of procrastination scale, failed to display any significant results, our maladaptive procrastination variable demonstrated a positive relationship with fear of failure. This finding is consistent with previous research, which studied the relationship between fear of failure and procrastination in its unidimensional model that focuses exclusively on the maladaptive aspects [10,12,63,113,114,115,116,117].

In addition, our maladaptive perfectionism variables yielded results that are consistent with previous research regarding these two concepts [65,118,119,120,121]. However, previous research has displayed a positive relationship between adaptive perfectionism and fear of failure, which was not replicated in the present study [65,119,120,121]. The differences observed in our data may be due to the use of parental criticism as our fear of failure scale. Studies investigating the relationship between parental criticism and adaptive perfectionism have found both a positive relationship, as well as no association between the two concepts [23,24,122]. Therefore, parental criticism may not be able to be used as a measure of fear of failure, and further research is needed to clarify this relationship.

### 4.4. Temporal Orientation

Unfortunately, adaptive procrastination did not have significant associations with any of the five time perspectives. However, part of our hypothesis was confirmed by our findings that maladaptive procrastination had a relationship with the past-negative, present-fatalistic, and present-hedonistic perspectives.

As predicted, the present study yielded significant results between adaptive perfectionism and the time perspectives. Adaptive perfectionism demonstrated a positive relationship with both the past-positive and future-oriented time perspectives. There has been very limited research on the relationship between the multidimensional model of perfectionism and the time perspectives originally proposed by Zimbardo and Boyd [71]. There was a single study conducted by Lagoutina [72] that was the first and only study so far to investigate a potential relationship between these variables. Some of the data from the current study replicated those found by Lagoutina [72]. Our measure of adaptive perfectionism demonstrated positive relationships with both past-positive, future-oriented, and present-hedonistic perspectives, all of which were also found by Lagoutina [72]. In addition, the present study replicated the findings that the past-negative perspective yielded no relationship with adaptive perfectionism, contrary to what was hypothesized. It has been theorized that having a young average age of participants in the study may lead to fewer past experiences to reflect on [72]. This explanation is consistent with the mean age of our sample population, which was 19.2 and consisted exclusively of undergraduate students.

However, there were some noticeable differences between the present study and Lagoutina’s [72] findings regarding maladaptive perfectionism. While our findings regarding adaptive perfectionism and the present-hedonistic perspective were consistent with Lagoutina’s [72], we found a strong positive relationship between our maladaptive perfectionism variables and the past-negative perspective. These findings contradict the lack of an association between these two concepts that Lagoutina [72] observed. While different scales were used to operationalize maladaptive perfectionism, this does not account for the difference in findings. The concern over mistakes and parental criticism subscales have been found to closely associate with socially-prescribed perfectionism, both accounting for distinct amounts of variance in scores on the socially-prescribed perfectionism subscale [23].

While the mean age of our sample may indicate fewer negative experiences, those who exhibit characteristics of maladaptive perfectionism are more likely to recall negative experiences and may be more likely to appraise an everyday event as negative. Previous research has found that maladaptive perfectionists actively process information across many situations, such as social interactions, generating interpretations of their personal experiences in a catastrophic manner [123,124,125]. Catastrophic automatic thoughts have been established as one of the many negative characteristics of maladaptive perfectionists [126,127]. Maladaptive perfectionists struggle with harsh self-evaluations and fear of failure, leading them to catastrophize everyday minor stressors, or even seemingly non-stressful events, that reflect their inability to complete a task in a manner that they view as acceptable or perfect [104,128,129]. These experiences do not need to be major life events in order to cause significant levels of distress. Minor stressors experienced throughout the day have been found to mediate the effects of major stressors on distress [130].

Moreover, past studies suggest that maladaptive perfectionists struggle with accepting their past, as well as identifying a purpose or meaning to their life [127]. Maladaptive perfectionists, characterized by heightened levels of an intolerance for mistakes and unrelenting self-scrutiny, may have trouble moving past events that they deem failures [131]. Due to these seemingly unforgivable failures, maladaptive perfectionists struggle to accept their past and tend to view it in a negative manner [127].

Additionally, maladaptive perfectionists are more likely to dwell on their past due to their tendency to ruminate. It has been discovered that rumination, when compared to worry, is considered more past-oriented, stemming from unresolved goals related to self-identity and understanding [132,133]. Previous studies have also found evidence linking rumination to maladaptive perfectionism in both college and community samples [134,135,136,137,138,139]. Maladaptive perfectionists are plagued by appraised negative experiences every day, ranging from their overly critical self-evaluations to their fear of failure influencing how they interpret events. The constant catastrophizing of events can be a heavy burden to bear, making it difficult for repetitive, intrusive, and self-degrading thoughts about past mistakes to be cast aside.

Furthermore, Lagoutina [72] found that high levels of maladaptive perfectionism, operationally defined by assessing socially-prescribed perfectionism from Hewitt and Flett [22], predicted a higher level of the present-hedonistic perspective. The data from the present study were inconsistent with these previous findings. We found that the present-hedonistic perspective had no associations with any of our maladaptive perfectionism variables, which includes the negative perfectionism, parental criticism, and concern over mistakes subscales. A possible explanation of the differences is the existence of three individual components that make up the present-hedonistic perspective. Due to the ambivalent nature of the present-hedonistic perspective, it has been broken down into three components: impulsivity/risk-taking, excitement seeking, and process orientation [140]. With the identification of these components, the present-hedonistic perspective is able to exist in both adaptive and maladaptive forms, with impulsivity representing the maladaptive form and process orientation reflecting the adaptive form [141]. It is possible that the scales used in the present study represented the more adaptive components of the present-hedonistic perspective, therefore causing a disparity between our results and those of Lagoutina [72].

Despite these differences, our findings regarding a relationship between maladaptive perfectionism and the present-fatalistic perspective were consistent with Lagoutina’s [72] findings. With a very limited number of studies investigating the relationship between perfectionism and temporal orientation, it is impossible to draw any concrete conclusions from this study. More research is needed to build a model for the relationship between these two concepts.

The importance of the time perspectives has been applied across many areas of research, the most notable area being the realm of academics, considering the population sampled in the current study consisted entirely of college students. The positive relationship between high levels of future orientation and several academic factors, such as academic achievement, academic engagement, and high college GPA, has been well documented [71,142,143,144,145,146]. Given the ability of the current study to replicate Lagoutina’s [72] findings regarding a positive relationship between adaptive perfectionism and future orientation, we can more confidently speculate that the academic success displayed by adaptive perfectionists may be partially explained by their tendency to maintain a future-oriented perspective [30,31,147,148,149,150,151,152].

### 4.5. Boredom Proneness

While adaptive procrastination failed to yield any significant results, the data regarding adaptive perfectionism was more telling. Due to the positive correlation with internal stimulation, adaptive perfectionists are able to generate their own source of motivation, which has been linked to many adaptive academic characteristics, such as high performance, engagement, and achievement [153,154,155]. Furthermore, the negative correlation between adaptive perfectionists and external stimulation shows that adaptive perfectionists do not require a constantly changing environment in order to stave off boredom. With both relationships considered, adaptive perfectionists are unlikely to experience high levels of boredom.

In regard to previous research, many factor analyses have been conducted on the original Boredom Proneness Scale [156]. From these studies, only two factors, internal stimulation, and external stimulation have consistently been replicated and are considered the best factors to illustrate the composition of the Boredom Proneness Scale [79,157,158,159,160,161,162]. Therefore, the lack of internal stimulation and the presence of external stimulation are both indicators of boredom.

Boredom is a widely researched construct, with several studies linking it to depression, anxiety, decreased life satisfaction, hopelessness, negative affect, fear, and decreased mindfulness [156,157,158,162,163,164,165,166]. Many of these negative characteristics have been applied to maladaptive perfectionism, providing support for our findings that adaptive perfectionism is not linked to boredom proneness [1,25,26,27,28,29,30,31,32,33,39].

On the other hand, the maladaptive procrastination variable, arousal procrastination, displayed the anticipated positive relationship with external stimulation. In addition, our prediction of avoidant procrastination having a negative relationship with internal stimulation was supported by our data. However, avoidant procrastination also yielded a positive relationship with external stimulation, which was not anticipated. Previous research has demonstrated a relationship between boredom proneness and avoidant and arousal procrastination. More specifically, avoidant and arousal procrastination have both been previously positively associated with external stimulation and negatively associated with internal stimulation [167].

The relationship between external stimulation and arousal procrastination discovered in the present study is consistent with previous research. External stimulation demonstrates a need for variety in the individual’s environment. The monotony of similar and repetitive tasks decreases their motivation, as well as increases their proneness to boredom [79]. Without an external environment that challenges them and maintains a certain level of variety, individuals high in external stimulation are not able to reach their optimal level of arousal and, therefore, are very likely to experience boredom [159,168,169,170,171]. Additionally, due to this constant need for variety, individuals who display external stimulation struggle with self-control [172].

Arousal procrastinators have previously demonstrated a positive relationship with impulsivity, sensation-seeking, and a present-hedonistic (risk-taking) attitude [15,173]. Arousal procrastinators intentionally delay tasks in pursuit of sensation-seeking. They put off tasks until the last minute, hoping that their self-imposed challenge of completing a task so close to the deadline will result in the attainment of some sort of rush or high [15,174,175]. This thrill-seeking experience of working under pressure is one method arousal procrastinators use to avoid boredom [176].

When compared, arousal procrastination and external stimulation display many similarities. Individuals exhibiting external stimulation have difficulties with self-control, which is also seen in arousal procrastinators and their impulsive behavior. In addition, arousal procrastination and external stimulation suffer from monotonous environments. Both require some type of variety or challenge in order to be actively engaged in a task.

The negative association between avoidant procrastination and internal stimulation also replicated previous findings. Internal stimulation is characterized by an individual’s perceived ineptitude to generate optimal motivation from within [79]. Individuals lacking internal stimulation struggle with inattention and low self-regulation, straining to keep themselves interested [157,162]. They lack the ability to self-generate activities or ideas that will keep their attention and keep them entertained. This ability to self-generate interesting activities or information can impact their proneness for boredom, as well as the regulation of their mood [177,178,179,180]. Furthermore, individuals lacking internal stimulation are not capable of formulating alternative solutions that would result in more active engagement with their goal or environment. Instead, they are fixated on a singular pathway to their intended goal or action, unable to engage in goal-directed action that would allow them to generate a more comprehensive plan for success [53,172].

Avoidant procrastinators act, or fail to act, based on the fear that, if they completed a task, their incompetence, and low skill level may be revealed and scrutinized by others [176,181]. Their low self-esteem and belief in their abilities lead them to put off a task, attempting to cope with the anxiety and threat from fear of failure [15,182]. By putting off a task, avoidant procrastinators are able to place blame on insufficient time if they fail to perform optimally as opposed to blaming their competence or abilities, therefore protecting their well-being [183,184]. In addition, avoidant procrastinators tend to avoid unappealing stimuli in their environments, thus avoiding active engagement in solving undesirable events [15,174,185,186]. Furthermore, avoidant procrastinators have been negatively associated with the present-fatalistic and future orientation time perspectives [173]. They tend to view their future, as well as life in general, as hopeless and predestined, believing they have no control over their present or future. Due to this passive view of control over their own life, avoidant procrastinators are able to protect their well-being and cope with their potential failures [173,183].

From this previous research, avoidant procrastination and internal stimulation seem to demonstrate a negative relationship. The fear of failure that avoidant procrastinators experience likely outweighs any type of internal stimulation, or interest, they are able to generate regarding a task. They are unable to generate enough motivation or interest to convince themselves to begin a task due to their excessive fear of negative evaluation, leading to inattention. Moreover, individuals lacking in internal stimulation fail to engage in goal-directed action, avoiding seeking alternative solutions to a singular problem, just as avoidant procrastinators do. Avoidant procrastinators also view their present and future as predetermined, further relating to a lack of internal stimulation through the lack of self-regulation or control over one’s experiences.

One finding from the current study that appears to contradict previous research is the positive relationship between avoidant procrastination and external stimulation. While there was a significant positive correlation between these two variables, it was not as strong as the correlation between arousal procrastination and external stimulation. The correlation observed between avoidant procrastination and external stimulation could be due to the heavy overlap that avoidant procrastination has with arousal procrastination. In the end, both variables measure procrastination, so they both exhibit the same relationships with variables such as impulsivity, inattention/distraction, moodiness, underachievement/disorganized, emotional difficulty, self-esteem, time perspectives, and several regret life domains [167,173,176,187].

However, Ferrari [167] discovered that both arousal and avoidant procrastination were positively correlated with external stimulation and impulsivity, a core characteristic of arousal procrastination. Yet, the correlation between avoidant procrastination and external stimulation in this study was not as strong as the relationship between arousal procrastination and external stimulation, similar to the findings of the present study. More research is needed to delineate the true relationship between external stimulation and avoidant procrastination.

### 4.6. Need for Affect

The present study provided evidence of a relationship between adaptive perfectionism and NFA approach, but unfortunately not adaptive procrastination. These findings contribute to the growing research on NFA and mental health. Studies have found that NFA approach is associated with positive emotions, such as joy and meaningfulness, as well as being linked to a healthy subjective well-being [188]. In addition, previous research has found correlations between NFA avoidance and poor mental health factors, even being linked to suicide risk, as well as job burnout [188,189,190,191]. However, contrary to these previous findings, our data indicated that NFA avoidance had positive associations with stress, depression, and anxiety, but the exact opposite relationship with maladaptive perfectionism. Interestingly, maladaptive perfectionism, strongly associated with these maladaptive characteristics, was negatively associated with NFA avoidance. It is unclear whether there is a mediating variable between these two variables, and future investigation is prompted to uncover this unknown variable that separates these two seemingly similar concepts.

### 4.7. Self-Efficacy and Euthymia

Adaptive procrastinators demonstrated one of the few relationships with adaptive perfectionists in this study through euthymia. Adaptive forms of both perfectionism and procrastination had a positive association with euthymia and self-efficacy. In contrast, euthymia and self-efficacy demonstrated an opposite association with the maladaptive forms of both perfectionism and procrastination. These findings from the present study further support existing data on the relationship between euthymia and the adaptive characteristics related to adaptive perfectionism [83]. Meanwhile, the link between euthymia and both adaptive and maladaptive forms of procrastination has yet to be established. Further research is needed to solidify the newly found relationship between these concepts.

### 4.8. Time Management

The present study failed to demonstrate a positive relationship between both adaptive procrastination and perfectionism and the moving ego metaphor, as well as maladaptive perfectionism and the moving time metaphor. However, correlations between the space-time metaphor variable (timeQ) and both decisional and avoidant procrastination illustrated the moving time metaphor. That is, individuals who exhibit the moving time metaphor, those who interpret the ambiguous question as “back” to Friday, reported higher levels of decisional and avoidant procrastination. Previous research regarding the time metaphors and procrastination are mixed. While some studies provide support for the findings of the present study, there are others that discovered conflicting evidence.

To begin with, there has been conflicting research on whether the moving ego or moving time metaphor is more related to the future-oriented time perspective, derived from the ZTPI. Richmond, Wilson, and Zinken [192] discovered that the moving ego metaphor was more closely related to the future-oriented perspective, while the moving time metaphor was associated with both the present-fatalistic and present-hedonistic perspectives. The data found in the present study support these previous findings. We discovered that maladaptive procrastinators demonstrated a relationship with the moving time metaphor. Since procrastinators have demonstrated a negative relationship with future orientation and a positive relationship with present orientation in past studies, the relationship between procrastination and the moving time metaphor is comprehensible [193,194,195,196,197,198].

However, Loermans and Milfont [199], who replicated the study conducted by Richmond, Wilson, and Zinken [192], asserted that, while their results did not yield any statistically significant correlations, those who exhibit the moving time metaphor are more closely related to the future-oriented perspective, while the present-oriented perspectives are more closely related to the moving ego metaphor. Other researchers support these findings, providing further conflicting evidence. Duffy and Feist [200] discovered that higher levels of procrastination, lower levels of conscientiousness, and higher levels of extroversion related to the moving ego metaphor. Building off of this previous research, Duffy, Feist, and McCarthy [201] used behavioral representations of conscientiousness and procrastination to demonstrate their relationships with either the moving ego or moving time metaphors. They found that those who were running late for an appointment and students who submitted their assignment close to the deadline represented the moving ego metaphor, while those who were early for appointments and students who submitted their assignment well ahead of the deadline represented the moving time metaphor [201]. Considering these previous studies, as well as others that have found evidence of a positive relationship between future orientation and conscientiousness, a negative relationship between conscientiousness and present orientation, and a negative relationship between conscientiousness and procrastination, there is an argument to be made that the moving ego metaphor, not the moving time metaphor, is indicative of procrastination [71,202,203,204,205,206,207,208,209,210,211,212,213].

To lend even more conflicting evidence, previous studies have investigated these metaphors and their relation to positive and negative affect, happiness, anxiety, and depression. They discovered the more adaptive concepts, including positive affect and happiness, were related to the moving ego metaphor, while the more maladaptive concepts, including negative affect, anxiety, and depression, were related to the moving time metaphor [192,214]. From the findings provided by these researchers, it can be deduced that maladaptive procrastinators, characterized by negative affect, anxiety, depression, and many other maladaptive characteristics, would be associated with the moving time metaphor.

With an abundance of research providing conflicting evidence, the relationship between procrastination and the time metaphors cannot be concluded. While the present study provides slightly more evidence regarding this relationship, future investigations are warranted.

### 4.9. Limitations

One limitation of the present study was the sample population from which we obtained our data. The participants of this study were comprised entirely of undergraduate students from one Midwestern university. Due to these limited population parameters, our data is not able to generalize to all other populations. Another limitation focused on the fact that the questionnaire completed by our participants was based entirely on self-reports. Furthermore, the results yielded from the present study are purely correlational, and therefore, no causal relationships can be determined. Moreover, the interpretations of the present data are limited due to the weak reliabilities of some of the scales used to measure certain variables, such as the positive perfectionism scale and the external stimulation scale.

Additionally, the present study relied heavily on conscientiousness to validate positive procrastination in the same way that positive perfectionism was established. Future research should divert from focusing on conscientiousness and consider other links that perfectionism and procrastination share in order to solidify the relationship between the two constructs and to determine with clarity the source of procrastination.

Moreover, there were some shortcomings in the variables tested and the instruments used to measure these variables. The most notable shortcoming was the lack of a direct measure for both motivation and self-regulation. While we included instruments that had self-regulatory and motivational aspects related to them, such as the boredom proneness scale, fear of failure scale, and the euthymia scale, our study would have benefited from having a more direct measure of motivation and self-regulation, since both of these constructs are considered key components of perfectionism and procrastination. With more measures of motivation and self-regulation included, an adaptive form of procrastination may be observed in the data.

For example, the concept of self-control plays an essential role in self-regulation and could have been utilized in the present study to further investigate the relationship between self-regulation, perfectionism, and procrastination. Preliminary links between these variables have been explored in conjunction with self-control. A previous study conducted by Achtziger and Bayer [215] analyzed the link between perfectionism and stress in a sample of freshman college students, discovering that self-control mediates this relationship. Additionally, they found a negative relationship between maladaptive perfectionism and self-control, as well as a positive relationship between adaptive perfectionism and self-control. Based on this, and previous research outlining a negative relationship between the unidimensional model of procrastination and self-control, the use of self-control as a measure of self-regulation could be very useful in studying self-control in the context of a multidimensional model of procrastination [215,216,217,218,219,220].

Previous researchers have utilized the conscientiousness subscale as an overt measure of self-control since conscientiousness often positively correlates with measures of self-control [221,222,223,224,225,226,227]. If conscientiousness is indeed able to be used as a proxy for self-control, the results from the present study would indicate that self-control has no relationship with adaptive procrastination. However, the present study would replicate the findings of Achtziger and Bayer [215], who found a positive relationship between self-control and adaptive perfectionism. The use of an overt measure of self-control, as opposed to direct self-reports, may prove useful in prompting future research into the concept of self-control and warrants further investigation [228].

## 5. Conclusions

In conclusion, the present study provides evidence that perfectionism and procrastination can only be linked by the negative characteristics they share through fear of failure, temporal orientation, external stimulation, and negative emotional aspects, such as depression, stress, and anxiety. They also demonstrate similar negative relationships with mostly adaptive constructs, including conscientiousness, satisfaction with life, internal stimulation, and euthymia. In addition, our data provide little evidence for the existence of an adaptive form of procrastination. However, the positive relationship exhibited between euthymia and adaptive procrastination gives hope that there may be more to the seemingly unidimensional model of procrastination that currently dominates research.

While the present study was able to replicate many previous findings regarding the multidimensional model of perfectionism, it also makes several new contributions to this area of research. Adaptive perfectionists display the majority of the characteristics anticipated by the dual-process model, providing further evidence of their adaptive nature. However, it is possible that individual experiences, especially those centered on achievement related-stressors, may have a dysfunctional impact on adaptive perfectionists, causing them to experience stress. Additionally, our study is one of only a few in existence that has attempted to identify the relationship between the multidimensional model of perfectionism and time perspectives. Adaptive perfectionists displayed a connection with the more adaptive time perspectives, the future orientation, past-positive, and present-hedonistic perspectives. Yet, they demonstrated no relationship with the past-negative perspective, a perspective considered to be maladaptive. This peculiar relationship requires further investigation and may reveal an aspect of adaptive perfectionists previously unknown.

## Figures and Tables

**Table 1:** Means, standard deviation, Cronbach’s alpha, and correlations.

Variable	M	SD	α	1	2	3	4	5	6	7	8	9	10
1. NP	18.35	4.75	0.80	-	−0.11	0.19 **	0.30 ***	0.60 ***	0.09	0.19 **	0.42 ***	0.31 ***	0.27 ***
2. PP	14.94	2.22	0.65	−0.11	-	0.45 ***	−0.25 ***	−0.06	0.05	−0.22 **	−0.23 ***	−0.17 *	−0.24 ***
3. SOP	61.04	10.71	0.80	0.19 **	0.45 ***	1.00	−0.04	0.30 ***	0.10	−0.39 ***	−0.13	−0.24 ***	−0.23 **
4. PC	9.19	3.69	0.83	0.30 ***	−0.25 ***	−0.04	-	0.47 ***	−0.05	0.19 **	0.20 **	0.21 **	0.31 ***
5. CM	23.28	7.13	0.90	0.60 ***	−0.06	0.30 ***	0.47 ***	-	−0.02	0.10	0.30 ***	0.18 *	0.19 **
6. COPS	28.07	8.28	0.91	0.09	0.05	0.10	−0.05	−0.02	-	0.12	−0.06	0.02	−0.02
7. GPS	46.41	8.99	0.82	0.19 **	−0.22 **	−0.39 ***	0.19 **	0.10	0.12	-	0.45 ***	0.73 ***	0.57 ***
8. DEC	14.48	4.34	0.89	0.42 ***	−0.23 ***	−0.13	0.20 **	0.30 ***	−0.06	0.45 ***	-	0.57 ***	0.54 ***
9. AVO	13.50	3.36	0.81	0.31 ***	−0.17 *	−0.24 ***	0.21 **	0.18 *	0.02	0.73 ***	0.57 ***	-	0.66 ***
10. ARO	13.97	4.12	0.79	0.27 ***	−0.24 ***	−0.23 **	0.31 ***	0.19 **	−0.02	0.57 ***	0.54 ***	0.66 ***	-
11. CON	41.85	6.31	0.82	−0.22 **	0.44 ***	0.45 ***	−0.26 ***	−0.23 ***	0.04	−0.62 ***	−0.47 ***	−0.58 ***	−0.57 ***
12. PN	3.13	0.71	0.73	0.39 ***	−0.13	0.01	0.31 ***	0.35 ***	−0.09	0.27 ***	0.31 ***	0.27 ***	0.28 ***
13. PH	3.49	0.54	0.84	−0.06	0.15 *	−0.01	−0.01	−0.13	−0.09	0.17 *	0.12	0.17 *	0.18 *
14. FUT	3.47	0.55	0.78	0.05	0.37 ***	0.53 ***	−0.12	0.03	0.01	−0.61 ***	−0.29 ***	−0.50 ***	−0.43 ***
15. PP	3.72	0.59	0.81	−0.13	0.36 ***	0.09	−0.43 ***	−0.23 ***	0.01	−0.03	−0.09	−0.04	−0.10
16. PF	2.77	0.66	0.76	0.31 ***	−0.19 **	−0.09	0.14	0.17 *	0.06	0.21 **	0.24 ***	0.13	0.22 **
17. PSS	24.52	5.51	0.84	0.59 ***	−0.12	0.13	0.39 ***	0.42 ***	−0.12	0.19 **	0.35 ***	0.34 ***	0.39 ***
18. CESD	18.38	4.41	0.84	0.42 ***	−0.16 *	0.03	0.44 ***	0.36 ***	−0.03	0.15 *	0.26 ***	0.27 ***	0.29 ***
19. STAI	12.38	3.88	0.83	0.32 ***	−0.19 **	0.02	0.28 ***	0.32 ***	−0.06	0.12	0.23 ***	0.21 **	0.28 ***
20. SWL	17.89	4.09	0.88	−0.28 ***	0.30 ***	0.17 *	−0.44 ***	−0.30 ***	0.06	−0.23 **	−0.29 ***	−0.17 *	−0.23 ***
21. timeQ	1.35	0.48	--	0.05	0.09	0.06	−0.05	−0.01	0.07	0.12	0.16 *	0.20 **	0.13
22. EUTH	5.20	2.11	0.72	−0.43 ***	0.24 ***	0.05	−0.34 ***	−0.34 ***	0.14 *	−0.22 **	−0.33 ***	−0.27 ***	−0.25 ***
23. AV	16.55	3.88	0.79	−0.24 ***	0.14	−0.03	−0.16 *	−0.26 ***	0.18 **	−0.02	−0.25 ***	−0.13	−0.22 **
24. AP	17.25	3.49	0.81	0.10	0.16 *	0.15 *	−0.04	−0.05	0.03	0.01	0.07	0.08	−0.03
25. IS	29.88	5.04	0.73	−0.23 ***	0.35 ***	0.28 ***	−0.21 **	−0.24 ***	0.10	−0.23 ***	−0.3 ***	−0.16 *	−0.14
26. ES	21.32	5.43	0.67	0.20 **	−0.17 *	−0.05	0.22 **	0.30 ***	−0.04	0.23 **	0.29 ***	0.17 *	0.20 **

Notes: All scales have N = 206; NP = Negative Perfectionism; PP = Positive Perfectionism; SOP = Self-Oriented Perfectionism (adaptive perfectionism); PC = Parental Criticism (maladaptive perfectionism); CM = Concern Over Mistakes (maladaptive perfectionism); COPS = Consequences of Procrastination (adaptive procrastination); GPS = General (Lay) Procrastination Scale; DEC = Decisional Procrastination; AVO = Avoidance Procrastination; ARO = Arousal Procrastination; CON = Conscientiousness; PN = Past-Negative; PH = Present-Hedonistic; FUT = Future Orientation; PP = Past-Positive; PF = Present-Fatalistic; PSS = Perceived Stress Scale; CESD = Center for Epidemiological Studies Depression Scale; STAI = State-Trait Anxiety Inventory; SWL = Satisfaction with Life; timeQ = Time Question; EUTH = Euthymia; AV = NFA Avoidance; AP = NFA Approach; IS = Internal Stimulation; ES = External Stimulation; * *p* < 0.05; ** *p* < 0.01; *** *p* < 0.001.
